# Immuno-histologic and histomorphometric evaluation of Angelica sinensis adjunctive to ß-tricalcium phosphate in critical-sized class II furcation defects in dogs

**DOI:** 10.1038/s41405-023-00150-y

**Published:** 2023-06-23

**Authors:** Dina W. Eldeeb, Ahmed M. Hommos, Maha R. Taalab, Samia S. Abd El Rehim

**Affiliations:** 1 Oral Diagnosis and Radiology Department, Faculty of Dentistry, Alexandria University, Alexandria, Egypt; 2 Faculty of Dentistry, Alexandria University, Alexandria, Egypt

**Keywords:** Health care, Anatomy

## Abstract

**Objective:**

The study evaluates the effectiveness of Angelica sinensis (As) adjunctive to Beta-tricalcium phosphate (β-TCP) bone graft in the management of induced critical sized class II furcation defects in dogs.

**Material and method:**

A randomized study design was conducted on the third and fourth premolars of six dogs. A total of twenty-four defects were surgically created. After reflecting a mucoperiosteal flap, twelve defects were filled with As granules mixed with β-TCP (Experimental group) while the other twelve defects were filled with β-TCP only (Control group) and both were covered by collagen membrane. At the fourth and eighth weeks, jaw segments were dissected and processed for immune-histological examination and histomorphometry analysis.

**Results:**

At four and eight weeks after treatment, experimental group showed a statistically significant increase in the height of newly formed interradicular bone (*p* = 0.001 and *p* = 0.0001 respectively), its surface area (*p* = 0.002 and *p* = 0.02 respectively), and the thickness of its trabeculae (*p* = 0.0001 and *p* = 0.001 respectively), when compared to control group. Moreover. alkaline phosphatase immunoreaction showed higher intensity in the osteoblast cells of experimental group compared to control group.

**Conclusion:**

As enhances periodontal regeneration and bone-formation when used in the management of furcation defects.

## Introduction

The periodontal disease is a complex, multifactorial, polymicrobial infection involving the destruction of tooth-supporting tissues including alveolar bone, periodontal ligament and cementum [1, 2]. Furcation involvement is the result of pathologic resorption of the supporting alveolar bone caused by the progression of periodontal disease. Though teeth presenting furcation involvement can be maintained for years under the acceptable conditions of care, the treatment of such defects always represents a clinical challenge [3].

Periodontal regeneration is the restoration of the tooth-supporting tissues structurally and functionally. Although a gain of clinical attachment level and bone fill are clinical indicators for successful regeneration, it is only by histological means the conclusive evidence of true regeneration can be achieved [4].

Bone grafting and barrier membranes are used together with biologically engineered tissue inductive proteins (e.g., growth factors, extracellular matrix proteins and bone morphogenetic proteins) resulting in osseous and periodontal regeneration [5].

Several bone grafting materials have reported to induce cell and bone tissue in-growth at the material surface [6]. Examples of commonly used grafts are autogenous grafts harvested from the patient’s iliac crest, mandibular ramus, or other intraoral sites, freeze‐dried bone allograft, demineralized freeze‐dried bone allograft, xenograft obtained from equine, porcine, or bovine bone, synthetic calcium sulphate, calcium phosphate, β tricalcium phosphate, hydroxyapatite and composite bioceramics [7, 8].

Barrier membranes act as a physical barrier for epithelial cell exclusion and for periodontal ligament and alveolar bone cells to repopulate the defect, thus allowing the desired regeneration of periodontal tissues [9]. The first generation of barrier membranes were the non-resorbable membranes polytetrafluoroethylene (PTFE), titanium reinforced ePTFE, high-density-PTFE, or titanium mesh [10]. In order to eliminate the need for second surgical procedure, the synthetic resorbable barrier membranes polyesters (e.g., polyglycolic acid, polylactic acid, polycaprolactone and natural barriers as tissue-derived collagen-based membranes from human skin, bovine Achilles tendon or porcine skin) have been developed as second-generation membranes [11, 12].

Chinese herbal medicine has a history of more than 2500 years and has accomplished remarkable effects in the clinical practice [13, 14]. Dong quai, dang gui or female ginseng are the commonly used terms for the root of Angelica sinensis. For thousands of years, Angelica sinensis has been part of the traditional Chinese, Korean, and Japanese medicine [15]. Researches have showed that it has various uses in the medical field as it acts as anti-inflammatory, immunomodulatory and anti-oxidative agent [16–18].

Angelica sinensis has been used to increase bone formation due to its both osteogenic and angiogenic effects, through its positive effect on growth factors such as vascular endothelial growth factor [19]. Moreover, Angelica sinensis polysaccharide may stimulate the osteogenic differentiation of rat bone marrow mesenchymal stem cells and promote bone regeneration under high glucose conditions by activating the Wnt/β-catenin signaling pathway [20]. Gao, et al. [21] provided the first evidence that Ferulic acid from Angelica sinensis can enhance the proliferation and differentiation of osteoblasts in vitro by expressing a number of osteogenic genes.

It was also reported that the topical application of a herbal formula containing Angelica sinensis as one of its compositions has shown ameliorative effects on the progress of periodontal breakdown through maintaining the integrity of periodontal structures, lowering the collagen degradation in gingival tissues and inhibiting alveolar bone resorption by the decreasing the osteoclastic activity [22].

The development of novel biomaterials for the treatment of severe furcation involvements is of great importance to clinicians. Therefore, this study was directed to investigate the regenerative potential of As herb for treating class II furcation defects. Since clinical analysis including probing depth, furcation entry, bleeding on probing and plaque index are often preformed in most of the human studies assessing different furcation treatment modalities, this study has considered instead the regenerative effect through the immunohistologic and histomorphometric analysis. The null hypothesis of this study proposed that there would be no significant difference in the regeneration of class II furcation defects in dogs with or without using As.

## Materials and method

### Materials

#### Study population

The research protocol involved six male mongrel dogs (Canis familliaris) which are the mostly used in experimental researches [23, 24].The protocol was approved by the institutional experimentation and Animal Ethical Committee of Alexandria University (IRBNO:00010556‐IORG0008839). For the care and the use of the animals in this study proper National Institutes of Health guide have been followed (National Institutes of Health Publications No.8023, revised 1978). The preclinical animal study conformed to the updated ARRIVE 2.0 guidelines.

#### Inclusion criteria and exclusion criteria

Systemically healthy male dogs, 18–24 months old and weighing approximately 10–18 Kg were included in the study. The dogs are matched regarding sex, age, weight, type of diet, and environmental housing conditions. The periodontal condition of the dogs was completely healthy with no any adverse sign of any gingival or periodontal disease. Dogs included in previous experimental study and dogs with any apparent illness or wounds were excluded.

#### Sample size estimation

The required sample size was estimated to be 12 defects per group using 5% alpha error, 80% power, and mean difference of 0.6 [±0.5] mm in bone height between experimental and control groups after 8 weeks which was based on a previous study [23]. Software Sample size is calculated using statistical software (G^*^power version 3.1.9.2; University of Düsseldorf, Germany, http://www.gpower.hhu.de/) [25].

#### Grouping and randomization

The study included twenty-four surgically induced critical sized class II furcation defects in the buccal surface of the right and left mandibular premolars (P_3_, P_4_). Twelve defects were assigned in the experimental group and were managed using As (Active Herb company, Suite E, San Diego), an osteoconductive allograft (Dental adbone; TCP, Medbone Biomaterials; Sintra, Portugal), and collagen membrane (Hypo Sorb; Bioimplon, Gießen, Germany). The other twelve defects acted as the control group with an osteoconductive allograft and collagen membrane only. In order to minimize the risk of cofounding, random allocation was done using a computer-generated random sequence of numbers to assign treatment status. Since each dog has four premolars, a random list was created to determine which teeth would receive the intervention by numbering each tooth and labeling dogs with IDs. This was done by giving each defect used in this study a number from 1 to 24 and using computer assisted software, a number of 12 defect was selected in each group.

#### Graft material

β-TCP is a radiopaque material with high degree of porosity and high mechanical strength (3.0 MPa). The porosity was 80% with pore size of 400 μm. The format was 0.1–0.5 mm granules in a 1 unit of 0.5 g. β-TCP has been used in control groups of similar studies testing the regenerative effects of different materials in grade II furcation defects [23, 24].

As is supplied in 1 bottle of 100 g of concentrated granules extracted from 330 g of the raw herbs with active ingredients of Ferulic acid ≥0.2 mg/g. The size of the granule ranges from 3 to 4 μm with granules Composition of 90% extract + 10% maltodextrin.

### Method

#### Surgical procedure

**Fig. 1:**
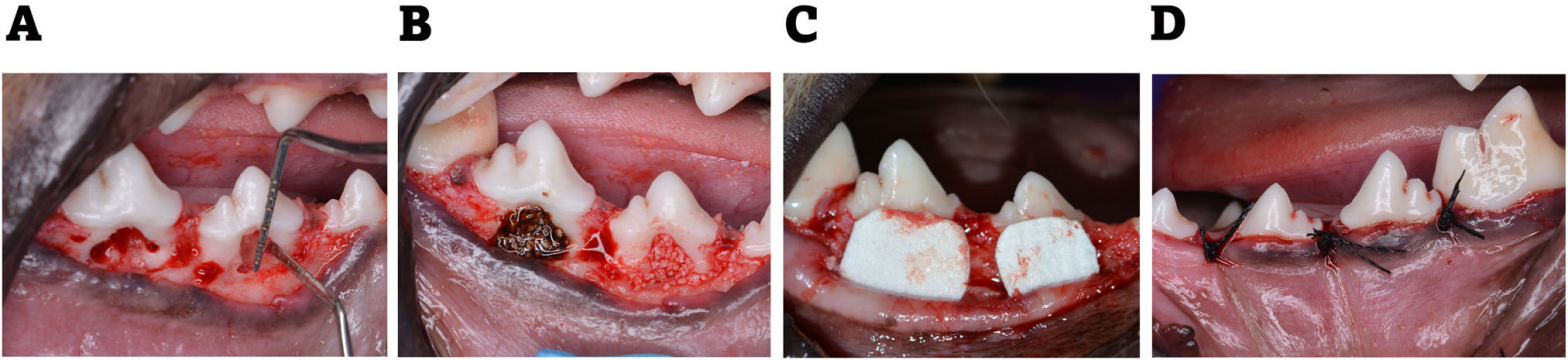
Illustration for the steps of the surgical procedures. **A** Measuring of the surgically created Class II furcation defects both vertically and horizontally in mandibular third premolar (P3) and mandibular fourth premolar (P4) Notice the reference notches on the inner sides of mesial and distal roots at apical end of the defect. **B** Placement of Angelica sinensis + ß‐TCP in P4 and only ß‐TCP in P3. **C** Buccal view of the collagen membrane adapted over the grafted defects. **D** Complete coverage of the managed defected by the use of interproximal interrupted sutures.

Proper sterilization of all the surgical instruments used in the procedures was ensured. The animals were generally anesthetized by sodium thiopental (Thiopental; Sandoz, Kundl, Austria)intravenous injection (13 mg/kg).1:100,000 epinephrine. Sulcular incisions at the buccal aspect of the mandibular third (P_**3**_) and fourth (P_**4**_) premolars in the right and left quadrants were then followed. It involved placing the blade vertically into the gingival sulcus, in such a way that the sharp edge of the blade remained against the tooth surface to prevent unnecessary damage to gingiva. The incision then followed the shape of the tooth. Mucoperiosteal flaps were fully reflected. A rotary 0.5 mm rounded carbide bur was used to create Grade II critical sized furcation defects of 3 × 4 at P3 and P4 for each dog [23, 26].The standardization of the defect size was carried out using 2 methods. First a rubber stopper was placed in the shank of the bur at the required cutting depth of 3 and 4 mms to guide where to stop the bone cutting. Second was that the defects dimensions were measured by the use of a periodontal probe both vertically and horizontally as shown in Fig. 1. The inter-radicular bone was removed under copious irrigation of sterile saline.

For the future histological examination, a reference point of the bone level surrounding the created defects at the time of the surgery was essential, therefore two reference notches were created on the mesial and distal root surfaces at the base of each defect [24]. Root planing by universal curette and root conditioning using Ethylenediaminetetraacetic Acid (MD Chelcream; META BIOMED, Cheongiu-si, South Korea) were carried out to the exposed root surfaces. Using the same calibrated spoon, equal scopes of As granules and β‐TCP were added to a dappen dish and mixed to achieve a ratio of (1:1) [23]. The defects of the experimental group were filled with As granules and β‐TCP mix, while the defects of control group were filled with β‐TCP alone. Trimming of the collagen membranes to the proper size extending 2–3 mm all around the created defects was followed by the application of the membranes to cover the graft filled defects. The used collagen membrane is known for lack of rigidity, good malleability during surgery, manipulation, and the capability to absorb blood. Collagen rapidly absorbs blood by generating artificial clot-like structure causing the underlying graft particles to be sticky. This means that once it contacts the blood, collagen provides a three-dimensional matrix via binding with a large number of platelets, causing platelet aggregation [27]. In addition to this membrane adhesive property, the defect size and so the membrane size were small and so required no sutures for fixation.

The flap was repositioned coronally to have a complete coverage of the defect area with no membrane exposure. The wound closure was achieved with simple interrupted suturing using 3-zero silk sutures (Mersilk; Ethicon Johnson & Johnson, Somerville, NJ, USA) (Fig. 1). These sutures were done interproximally; that is between P3 and P4, mesial to P3 and distal to P4. The simple interrupted technique is achieved by first penetrating the buccal gingiva, crossing the wound, and exiting the lingual tissue. A loop is created, and the suture thread is tied off at the buccal entry point. These sutures ensured proper approximation of wound edges with no tension and guaranteed complete wound closure.

#### Postoperative care

The dogs were given intramuscular injection of ampicillin (1 g) (Alzental; Eipico, Tenth of Ramadan City, Egypt) and Ibuprofen (600 mg) (Brufen; Abbot, Mannheim, Germany) in the first day. For the following week, the dogs received the medication mixed with their food. Throughout the study period, the dogs were fed soft diet to minimize the possibility of local trauma at the site of operation. Sutures were removed after 10 days.

The animals were kept in collective kennels, one per kennel, under the normal atmospheric temperature. Good ventilation and light/dark cycle (12/12 h) were maintained with free access to standard food and water. The standard diet regimen was replenished daily throughout the experimental period. The animals were routinely checked for weight loss, gingival and soft tissue inflammation. Regular teeth cleaning with dog’s toothbrush was preformed for the entire study period for effective plaque control

#### Animal euthanasia

Three dogs were euthanized at 1 month postoperatively and the other three dogs after 2 months. These time points were according to a study conducted by Afifi et al. investigating the regenerative effect of another herb in furcation defects on dogs [23]. The euthanasia involved the use of overdose intravenous injection of concentrated thiopental sodium (Thiopental; Sandoz, Kundl, Austria).

#### Histological procedure

After euthanasia, the mandibles were dissected out and the jaw segments containing the operated teeth were separated and fixed in 10٪ neutral buffered formalin. They were decalcified in multiple baths of 5% trichloroacetic acid and processed to obtain 5 microns thickness mesiodistal serial sections which were stained with Hematoxylin and Eosin (H&E) and Gomori trichrome and examined by light microscope to evaluate the regenerative potential in the defects and perform the morphometric analysis. Concealment of group allocation from the researcher preforming the histological examination ensured a single-blind study and made the results of the study less likely to be biased.

#### Histomorphometric quantitative analysis

Histologic photomicrographs were quantitatively analyzed using image J 1.46 r software (Image J version 1.46r; NIH, Bethesda, [MD], USA, https://imagej.nih.gov/ij/download.html) [28]. Measurements were performed in mm or fractions of mm.

Three variables were analyzed:

1. Height of newly formed bone in the interradicular region. This is the distance from the most coronal point of the formed bone till a line crossing between the two notches on the mesial and distal roots at the most apical end in mm. Standardized magnification of ×40.
2. Mean percentage of the newly formed bone surface area. Standardized magnification of ×100.
3. Thickness of newly formed bone trabeculae in mm. Standardized magnification of ×100.

From each of the 24 specimens of the defects, two sections were obtained and hence a total of 48 sections were examined (24 sections from the control group, 12 for each observation period and the other 24 sections from the experimental groups, 12 for each observation period). From each of the chosen sections, one standardized image for specific same structures needed for quantification was taken then measurements were recorded and statistically analyzed. All the morphometric analysis was carried out by one pre-calibrated investigator (SM). Each measurement was taken 2 times, two days apart, and the mean was then recorded (Intra-examiner correlation coefficient = 0.86,95% confidence interval = 0.81–0.92).

#### Statistical analysis

Data were tested for normality using the Shapiro–Wilk test and Q-Q plots. Normal distribution was confirmed for bone height, surface area, and thickness, therefore, the intervention in the two groups was analyzed with unpaired t-test. Tests were two-tailed, and the significance level was set at a *p*-value of 0.05. Data were analyzed using dedicated statistical software (SPSS version 28.0; IBM, Armonk, NY, USA, https://www.ibm.com/spss).

#### Interpretation of immunoreactivity

Alkaline phosphatase positive osteoblasts were traced and assessed in the different groups included in the study as intense, strong, moderate, and weak, depending on morphological descriptive scale. Samples were considered positive when the cells consistently had cytoplasmic color change (brown, black).

## Results

### Clinical observations

During the postoperative period, no clinical adverse reactions were noted. The dogs were clinically healthy and did not exhibit any sign of site infection or wound contamination.

### Histological results

4 weeks observation period:

1. Experimental group
  Formation of regenerative bone was noted in most of the sections obtained from different samples of experimental group. However, the formed bone did not fill the defects completely, but some areas in the central regions accommodated β-TCP particles with some patches of As, (Fig. 2A, B). Active figures of Angiogenesis were traced in the regenerating PDL, (Fig. 2C).

**Fig. 2:**
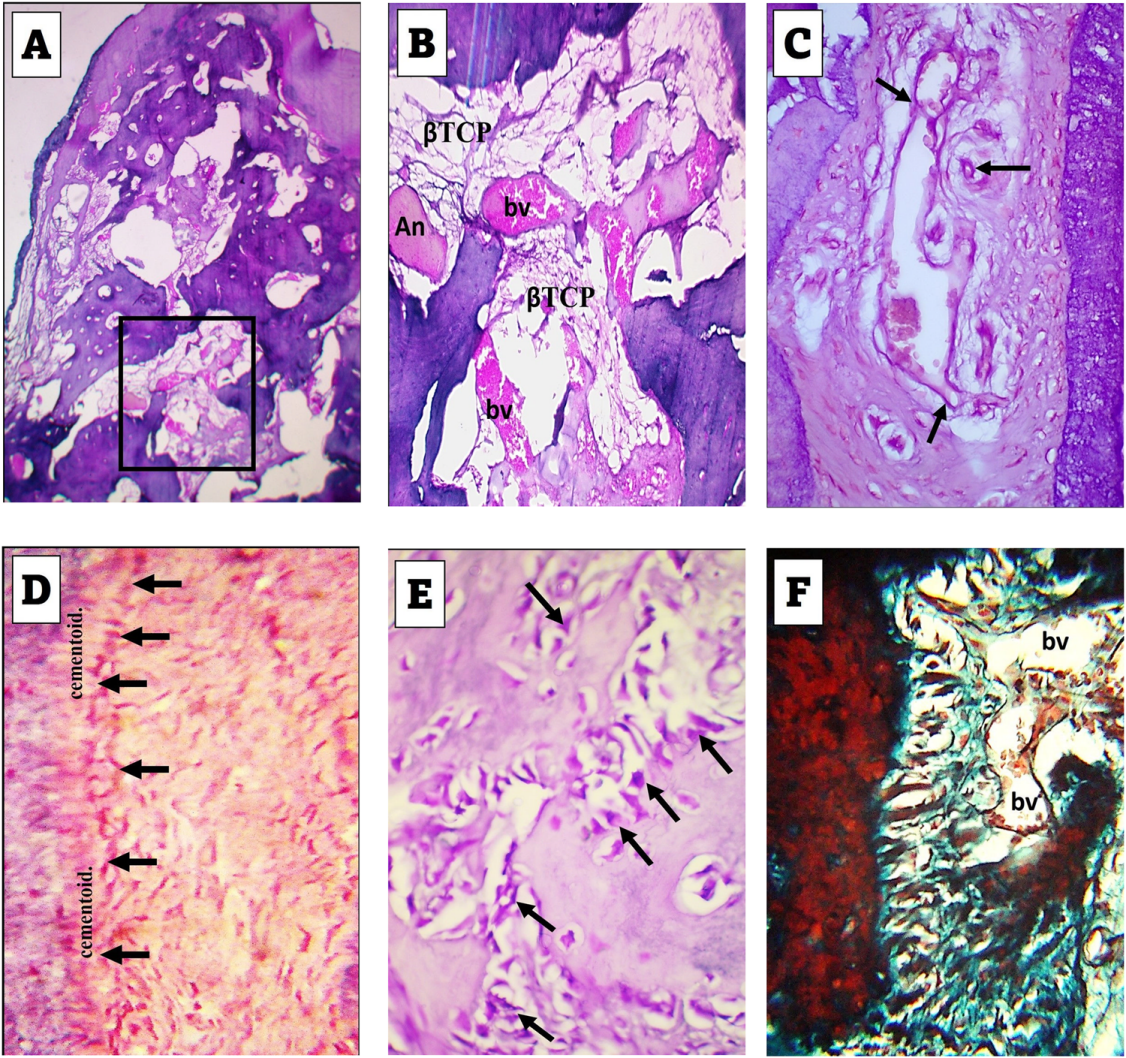
Results of 4 weeks experimental group. **A** Formation of regenerating bone in most of the defect regions with considerable spaces containing βTCP and some figures of Angelica sinensis (An). H&E stain: ×40. **B** Higher magnification of the boxed area in A revealing the proliferating blood vessels (bv). H&E stain: ×100. **C** Angiogenesis (arrows) in the regenerating PDL. H&E stain: ×400. **D** Cementoblasts (arrows) adjacent to a thin ribbon of cementoid. Note the random distribution of fibroblasts in the regenerating PDL. H&E stain: ×400. **E** Voluminous osteoblasts (arrows) adjacent to forming bone trabeculae. H&E stain: ×400. **F** Insertion of the newly formed fibers in the regenerating bone. Note the state of PDL regeneration and blood vessels (bv). Trichrome stain: ×400.
  At higher magnification of examination, prominent cementoblast cells were traced on the border of the forming cementum and were separated from it by a layer of cementoid, (Fig. 2D). Also, voluminous osteoblasts could be traced bordering the newly formed trabeculae, (Fig. 2E). In trichrome stained sections, the regenerating fibers of the forming PDL could be traced inserting in the forming alveolar bone, (Fig. 2F).
2. Control group
  The histological picture differed from that of the experimental group with less amount of formed bone which was especially deficient at the most coronal boundary of the defect. The regenerating bone consisted of irregular thin trabeculae with intervening spaces containing considerable amount of β-TCP particles, (Fig. 3A, B).

**Fig. 3:**
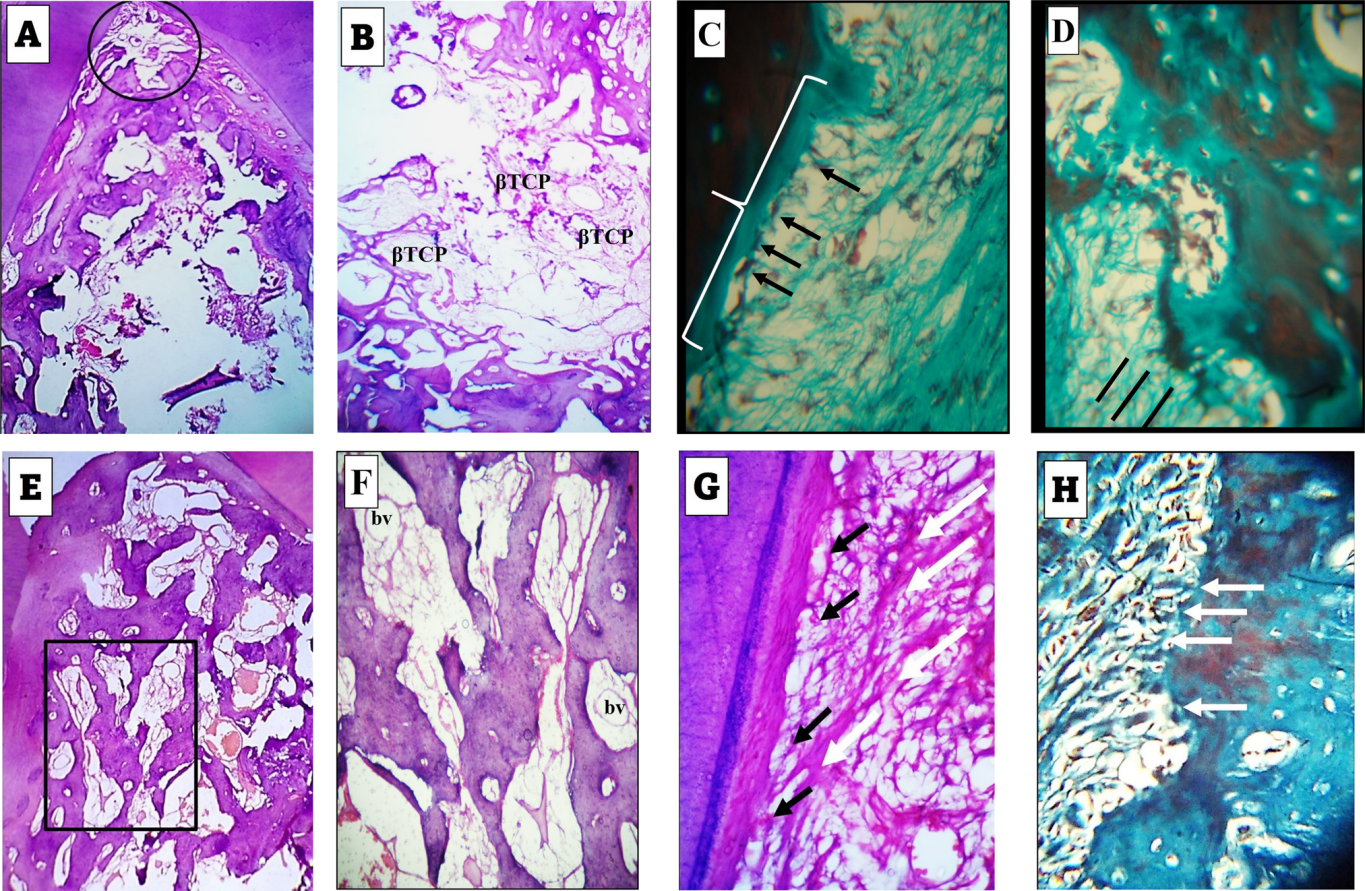
A-D figures of control group of 4 weeks observation period, E-H figures of control group of 8 weeks observation period. **A** Formation of less amount of regenerating bone than that of the experimental group of 4week period with absence of bone formation in the most coronal part of the defect (circle). H&E stain: ×40. **B** Thin bone trabeculae formed near the basal part of the defect with considerable spaces containing βTCP. H&E stain: ×100. **C** Flattened cementoblasts (arrows) differentiating adjacent to the border of the forming cementum (brace). Trichrome stain: ×400. **D** Early insertion of the forming PDL fibers in the newly forming bone (straight black lines). The PDL fibers exhibit a random course for new arrangement in the region spanning cementum and bone. Trichrome stain: ×400. **E** Formation of moderate amount of regenerating bone less than that of the 8 weeks experimental group. H&E stain: ×40. **F** Higher magnification of the boxed area in (**E**) revealing βTCP particles and blood vessels (bv). H&E stain: ×100. **G** Slightly flattened cementoblasts (black arrows) differentiating adjacent to the border of the forming cementum. The fibers of the forming PDL exhibit remodeling features with thickness variation and specific directivity towards cementum surface (white arrows). Trichrome stain: ×400. **H** Insertion of the forming PDL fibers in the newly forming bone (arrows). Note that these fibers are not aligned in bundle aggregates and cannot be followed for long distance into the bone. Trichrome stain: ×400.
  Towards the periphery of the defect, the forming fibers of the PDL appeared thin, discontinuous, and exhibited random course for insertion in either of the regenerating alveolar bone or cementum, the latter was bordered by flattened cementoblasts. These randomly arranged fibers enclosed flattened fibroblasts, (Fig. 3C, D).

8 weeks observation period:

1. Experimental group
  The histological findings were the best among all of the included groups in this study. Bone formation extended to a higher level coronally than its level at the 4 weeks observational period. The formed bone filled almost most of the defect, (Fig. 4A). The formed trabeculae were thicker than those seen in the 4 weeks observation period and exhibited a well intercommunicating pattern. A considerable density of blood vessels was traced among these trabeculae, (Fig. 4B).

**Fig. 4:**
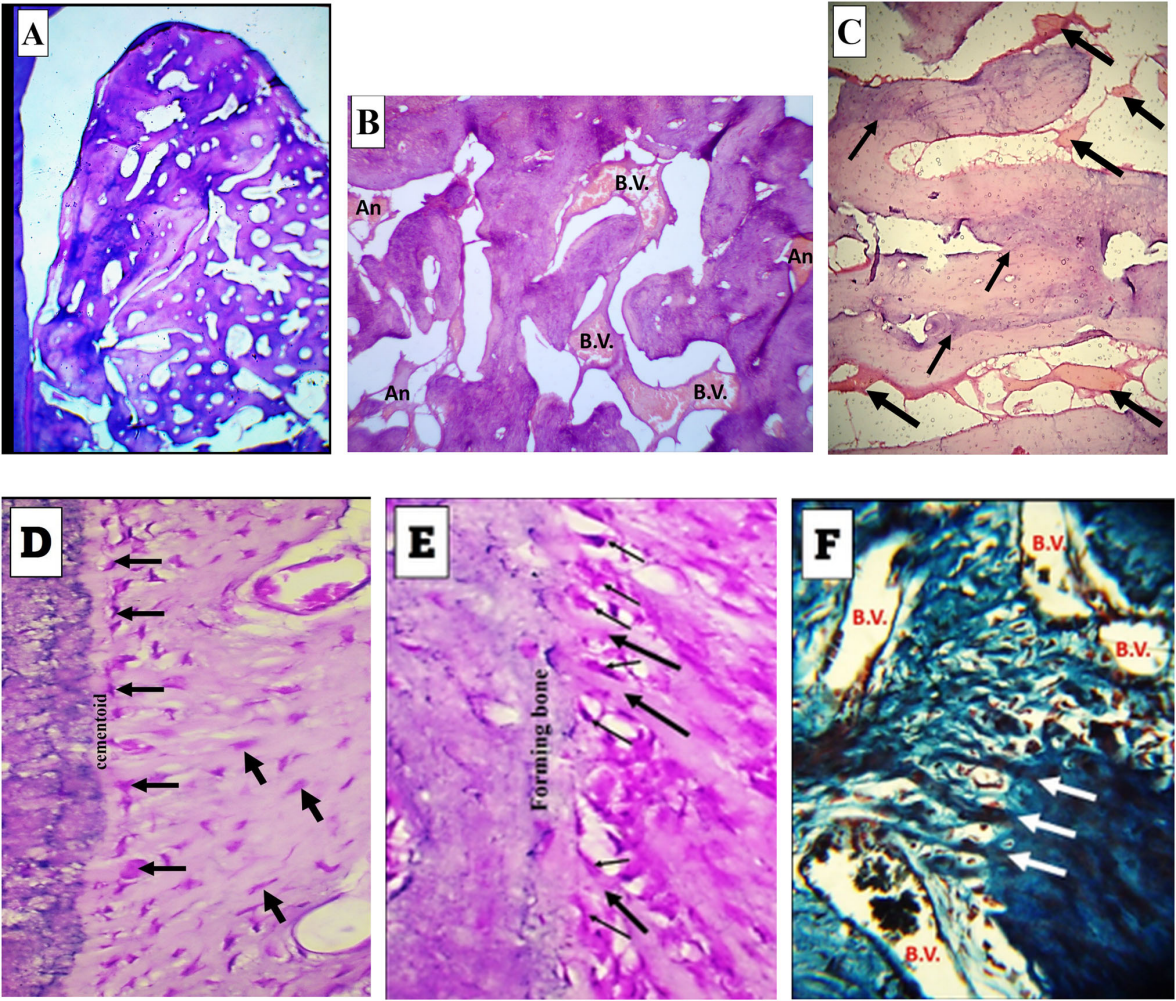
Results of 8 weeks experimental group. **A** Formation of dense regenerating bone in most of the defect regions extending from the bifurcation till the base. H&E stain: ×40. **B** High power view of a part of the central region of one of the defects. Note the thickness of the trabeculae, the blood vessels (bv) and Angelica patches (An). H&E stain: ×100. **C** Predominance of remodeling figures in most of the formed trabeculae (thin arrows) and the appearance of Angelica patches between the growing and remodeling trabeculae (thick arrows). H&E stain: ×100. **D** Well-aligned and organized cementoblasts (thin arrows) adjacent to a thin ribbon of forming cementoid. Note the organized fashion of fibroblast distribution in relation to the regenerating fibers of the PDL (thick arrows). H&E stain: ×400. **E** Well aligned and organized osteoblasts (thin arrows) adjacent to the forming bone. Note the fiber insertion in the regenerating bone (thick arrows). H&E stain: ×400. **F** Thick bundles of the forming PDL fibers are seen inserting deeper through the regenerating bone. Many blood vessels are evident (bv). Trichrome stain: ×400.
  In this group, the regenerating bone characterized by outstanding feature of remodeling revealed by different levels of bone stainability. The remodeling figures predominated and occurred in closed association with patches of As (Fig. 4C). Adjacent to both of the regenerating cementum and bone, the cementoblast and osteoblast cells appeared sizable and highly organized in relation to either tissue than their appearance in association with the other groups, (Fig. 4D, E). In trichrome stained sections, the PDL fibers formed thick bundles and could be traced for deeper levels in the regenerating alveolar bone and surrounded by many blood vessels, (Fig. 4F).
2. Control group
  The defects exhibited moderate degree of filling with bone trabeculae of variable size and separation. Moderate figures of blood vessels and β-TCP particles were seen among these trabeculae, (Fig. 3E, F) Towards cementum, Collagen fibers of regenerating PDL exhibited strong appearance of remodeling and thickness variation. Cementum reformation and regeneration were also evident, (Fig. 3G). Collagen fibers of regenerating PDL were inserted in the regenerating bone, but most of them did not exhibit the bundle aggregation which was seen in the experimental group of the same observational period, (Fig. 3H).

### Results of histomorphometry analysis

The histomorphometry assessment of the three measured parameters (height of the formed interradicular bone, percentage of bone surface area, and thickness of bone trabeculae) at the two observation periods showed comparable results in both groups with greater values in As experimental group. (Table 1).

**Table 1:** Numerical comparison between the study and control groups at the 2 observational periods as regard to the three parameters of interest.

Observational Period	Height of newly formed interradicular bone (mm)		Percentage of bone surface		Trabecular thickness (mm)	
	Control Group	Experimental Group	Control Group	Experimental Group	Control Group	Experimental Group
(mean ± SD)	2.51 ± 0.15	3.03 ± 0.09	44.63 ± 7.94	58.88 ± 13.92	0.23 ± 0.05	0.468 ± 0.08
Median	2.4	2.98	47.45	63.56	0.205	0.43
Min-max	2.3–2.7	2.93–3.22	30.05–56.16	45.90–72.62	0.19–0.358	0.32–0.58
* P* value	0.001		0.002		0.0001	
* t*-value	1.83		1.812		1.795	
2 months						
(mean ± SD)	2.95 ± 0.25	3.57 ± 0.11	64.70 ± 7.75	78.35 ± 5.76	0.37 ± 0.05	0.57 ± 0.11
Median	2.87	3.55	68.29	74.97	0.36	0.55
Min-max	2.48–3.32	3.41–3.71	49.61–79.31	69.28–86.96	0.29–0.47	0.40–0.73
* P* value	0.0001		0.02		0.0001	
* t*-value	1.82		1.813		1.786	

*P* value ≤ 0.05 is considered significant value.

As referred to Table 1, the height of newly formed interradicular bone in the experimental group was significantly higher than that of the control group after 1 month (mean = 3.03 ± 0.09 and 2.51 ± 0.15 mm respectively) with *p* = 0.001 and after 2 months (mean = 3.57 ± 0.11 and 2.95 ± 0.25 mm respectively) with *p* = 0.0001.

The percentage of bone surface area of the As/ ß‐TCP experimental group was significantly higher than that in the control group after 1 month (mean = 58.88 ± 13.92 and 44.63 ± 7.94% respectively) with *P* = 0.002 and after 2 months (mean = 78.35 ± 5.76 and 64.70 ± 7.75% respectively) with *P* = 0.02.

After 1 month the newly formed trabecular bone thickness in the experimental group was significantly higher than that in the control group (mean = 0.468 ± 0.08 and 0.23 ± 0.05 mm respectively) with *P* = 0.0001. The difference was also significantly higher at 2 months period (mean = 0.57 ± 0.11 and 0.37 ± 0.05 mm respectively) with *P* = 0.0001.

### Results of immunohistochemistry

**Fig. 5:**
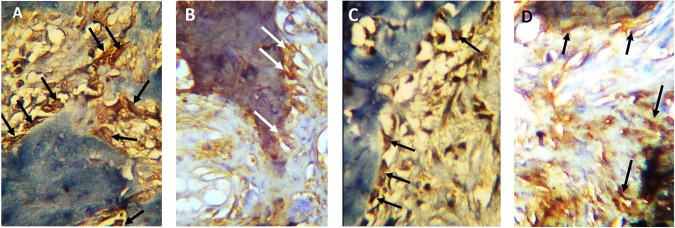
Alkaline phosphatase immunoreactivity. Arrows in all four figures point to osteoblast cells adjacent to the regenerating bone trabeculae magnification: ×400. **A** At 4 weeks experimental group, intense expression of alkaline phosphatase enzyme. **B** At 4 weeks control group moderate expression of alkaline phosphatase enzyme. **C** At 8 weeks experimental group, strong expression of alkaline phosphatase enzyme. **D** At 8 weeks control group, moderate expression of alkaline phosphatase enzyme.

The osteoblastic activity of the experimental and control groups was further evaluated by bone alkaline phosphatase intensity [29]. At 4 weeks observation period, experimental group showed an intense ALP reaction predominating the voluminous osteoblast cells bordering the regenerating trabeculae of the defects (Fig. 5A). The control group, however, revealed osteoblast cells exhibiting a moderate intensity of ALP activity, (Fig. 5B). At 8 weeks observation period, experimental group showed a strong ALP reaction in osteoblast cells, (Fig. 5C). Within the same time interval, osteoblast cells of control group revealed a moderate ALP labeling, (Fig. 5D).

## Discussion

This study was conducted to investigate if Angelica sinensis herb could be effective in managing surgically induced class II furcation defects as it can stimulate periodontal regeneration and enhance the bone formation. Randomization of the induced defects on whether to receive Angelica sinensis and β‐TCP or β‐TCP alone has excluded the environmental and biological tissue factors that are present between different animals. Any difference between the groups could therefore be referred to the regenerative effect of Angelica sinensis. Few if any study has measured its regenerative osteogenic effect when used intraorally on large-sized animal (dogs). To the best of our knowledge, this study is the first one to include the application of Angelica sinensis in the management of class II furcation defects in experimental animals. The literature does not contain similar studies on human.

The findings obtained in this study have rejected the null hypothesis which was proposed before preforming the experiment as it showed that As when mixed with β‐TCP results in more bone and periodontal regeneration compared to β‐TCP alone.

The increased bone formation in the experimental group compared to control group as shown by the histological and histomorphometry quantitative analysis has reflected the osteo-inductive potential of Angelica sinensis. This was also revealed in an in vivo study by Xie X, et al. [30] in which Angelica polysaccharide has promoted the same effect on rats mesenchymal stem cells by regulation of long non-coding RNA H19.

Moreover, the high regenerative ability of Angelica sinensis can be contributed to its anti-inflammatory effect which was proved by Kim YJ, et al. [31] who reported that Angelica sinensis root water extract has anti-inflammatory effect on lipopolysaccharide-induced mouse macrophages. Other studies reported that the volatile oil of Angelica sinensis has shown a potential anti-inflammatory mechanism by plasma metabolomics approach that has significantly inhibited systemic inflammatory response mediated by acute local stimulation [32, 33].

Apart from the previously reported strong stimulation of osteogenesis, the present study proved that Angelica sinensis exerted a regenerative effect on cementum and PDL regeneration. The histological sections of the experimental groups showed increased cementoblast-like cells along the uniformly deposited cementum layer into which Sharpey’s fibers were inserted and fibroblast-like cells in a highly vascular PDL space. The thick fiber bundles of PDL in experimental group of 8 weeks reflects the stimulating effect of Angelica sinensis on fibroblast cells. These results are in accordance with the results of studies by Zhao H et al. [34, 35] in which SBD.4A-a defined multicomponent preparation of Angelica sinensis has stimulated inflammatory healing responses mediated by human periodontal ligament cells, through the proliferation of fibroblast cells which in turn increased the amount of produced hyaluronic acid.

Another considerable finding observed in the histological sections of Angelica sinensis test groups were the greater density of newly formed blood vessels as compared to the control groups. Angelica sinensis exerted an evident effect on the proliferation and dilation of blood vessels revealed by the close association between them in most of the sections examined from the two experimental groups. The current study results are in agreement with the results of a study which proved the angiogenic potential of Angelica sinensis and described how this angiogenic potentiality of Angelica sinensis can treat ischemic strokes [36]. Meanwhile, the investigation by Lam HW et al. [37] revealed this angiogenic effect on human endothelial cells both in vitro and in vivo.

When comparing different modalities used in the treatment of Class II furcation defects in dogs, Angelica sinensis shows comparable results as regard to percentage of bone surface area. Deliberador TM et al. [38] investigated the effect of autogenous bone graft with or without a calcium sulfate barrier. After three-month observation period, the percentage of bone surface area for autogenous bone graft with and without a calcium sulfate barrier were 59.85( ± 20.90) and 64.95( ± 15.75) respectively whereas for Angelica/ β-TCP was 58.88( ± 13.92) after 2-month observational period only. Another study by Simsek et al. [39] comparing the effect of mesenchymal stem cells (MSCs) and autogenous cortical bone graft (ACB) in the treatment of class II furcation defects in dogs revealed percentage of new alveolar bone area after 8 weeks of 84.60 ( ± 4.85) for ACB and for 80.47 ( ± 8.23) for MSCs. These results are comparable to the result obtained in the current study in which the percentage of the formed bone surface area after 8 weeks was 78.35 ( ± 5.76).

ALP expression is one of the early markers for osteogenesis [40]. In this study, we analyzed the reaction intensity of tissue ALP. It showed increased ALP immunoreactivity at 8 weeks’ time point, suggesting its critical role in bone matrix calcification rather than osteoblastic differentiation only.

Several studies have investigated the regenerative effect of β-TCP in the treatment of furcation defects. The results reported that β-TCP were successful at new bone formation and periodontal healing [41–43]. In contrast, Stavropoulos et al. [44] concluded that although β -TCP can result in probing depth reduction and clinical attachment gain, it does not enhance the regeneration of cementum, periodontal ligament, and bone. Thus, the superior outcomes in experimental groups are only allocated to As regenerative features rather than β –TCP.

Through the study model in this investigation, As has demonstrated increased regenerative capability through measuring the amount of the formed bone. In further researches, it is recommended to analyze the surface area of the proliferating blood vessels and investigate the expression of Vascular Endothelial Growth Factor and Fibroblast Growth Factor in defects managed by As. A more precise evaluation of the regenerative potential of As on the cementum can be achieved by investigating the thickness of newly formed cementum. Furthermore, more resorbable and easily tailored alloplastic materials such as Brushite or Monetite should be considered instead of β –TCP.

## Conclusion

The management of furcation defects with As and β-TCP leads to better bone formation as regard to the rate, quality and quantity of new bone when compared to the use of β-TCP alone. As is a novel biocompatible material with a highly regenerative potential as confirmed by the immunohistologic and histomorphometric analysis.

## Data Availability

The data set used or analyzed during the current study are available from the corresponding author upon reasonable request.
